# Comparative efficacy of continuous versus intermittent administration of furosemide in acute heart failure: an updated systematic review and meta-analysis of 22 RCTs

**DOI:** 10.3389/fcvm.2026.1792136

**Published:** 2026-06-22

**Authors:** Juan Cai, Danpeng Wang, Ling Yang, Ye Yuan

**Affiliations:** 1 Department of Cardiology, Nantong Hospital of Traditional Chinese Medicine, Nantong, China; 2 The Second Affliliated Hospital of Chengdu University of Traditional Chinese Medicine, Chengdu, China; 3 Hospital of Chengdu University of Traditional Chinese Medicine, Chengdu, China; 4 Sichuan Integrative Medicine Hospital, Chengdu, China

**Keywords:** acute heart failure, furosemide, meta-analysis, randomized controlled trials, systematic review

## Abstract

**Introduction:**

Acute heart failure is defined as the rapid or gradual onset of signs and/or symptoms of heart failure severe enough to warrant unplanned hospital or emergency department care. As of 2019, an estimated 56.2 million individuals across 204 countries and territories were living with heart failure; data gaps suggest the true burden is likely higher. The global number of HF cases doubled from 27.2 million in 1990 to 56.2 million in 2019, with a parallel doubling observed in both sexes. Intravenous loop diuretics—predominantly furosemide—constitute the cornerstone of decongestive therapy, yet the optimal dosing strategy is still debated. Previous studies have established the framework for this field but yielded conflicting conclusions. In addition, those studies had some limitations. Therefore, we conducted an updated systematic review and meta-analysis that identified and included all RCTs comparing continuous infusion with intermittent bolus furosemide in adult patients with acute heart failure or NYHA class III–IV symptoms.

**Methods:**

We conducted a systematic literature search in PubMed, Embase, the Cochrane Library, CNKI, Wan Fang, and VIP databases for relevant studies published up to 1 October 2025. RCTs comparing continuous infusion with intermittent bolus furosemide in adult patients with acute heart failure or NYHA class III–IV symptoms were included. Primary endpoints were all-cause mortality and freedom from congestion. Secondary endpoints were weight loss, 24-h urine volume, length of hospital stay, 72-h urine volume, post-treatment BNP, edema resolution time, time to dyspnea improvement, post-treatment LVEF, and post-treatment LVEDD.

**Results:**

A total of 22 RCTs with 2,633 participants were included. The intermittent intravenous (iIV) arm showed a numerical trend toward all-cause mortality without reaching statistical significance (low evidence quality) compared with the continuous intravenous (cIV) arm (RR: 1.36, 95% CI: 0.96–1.94, *p* =  0.08). The cIV arm demonstrated significant improvements in freedom from congestion (moderate evidence quality) compared with the iIV arm (RR: 1.42, 95% CI: 1.06–1.91, *p* = 0.02). In addition, the cIV arm showed significant improvements in weight loss (moderate evidence quality), 24-h urine volume (low evidence quality), length of hospital stay (low evidence quality), edema resolution time (low evidence quality), time to dyspnea improvement (low evidence quality), post-treatment LVEF (low evidence quality), post-treatment LVEDD (low evidence quality), and post-treatment BNP level (moderate evidence quality).

**Discussion:**

Compared with intravenous bolus furosemide, continuous infusion was associated with faster relief of congestion, greater weight loss, and greater reduction in post-treatment BNP levels (moderate evidence quality). However, no significant advantage was demonstrated for the remaining outcomes or long-term survival, as judged by the quality of evidence and effect estimates.

**Systematic Review Registration:**

https://www.crd.york.ac.uk/PROSPERO/view/CRD420260223267, identifier CRD420260223267.

## Introduction

Acute heart failure (AHF) is defined as the rapid or gradual onset of signs and/or symptoms of heart failure (HF) severe enough to warrant unplanned hospital or emergency department care (1). As of 2019, an estimated 56.2 million individuals across 204 countries and territories were living with heart failure; data gaps suggest the true burden is likely higher. The global number of HF cases doubled from 27.2 million in 1990 to 56.2 million in 2019, with a parallel doubling observed in both sexes (2). Despite advances in chronic HF therapy, post-discharge re-hospitalization and 6-month mortality after an AHF episode remain as high as 30% and 15%, respectively (3, 4). Intravenous loop diuretics—predominantly furosemide—constitute the cornerstone of decongestive therapy, yet the optimal dosing strategy is still debated (5).

Two conceptual approaches have evolved over the past four decades: continuous intravenous and intermittent intravenous administration. Proponents of cIV argue that sustained delivery maintains a constant threshold concentration above the tubular “ceiling,” thereby promoting steadier natriuresis, avoiding post-receptor rebound, and reducing neurohormonal activation, ototoxicity, and electrolyte shifts (6–8). Conversely, advocates of iIV highlight logistical simplicity, lower drug and fluid load, preserved diuretic efficacy through repeated peak concentrations, and the possibility of intermittent “diuretic holidays” that may permit tubular recovery (9, 10).

Previous studies have laid an important foundation for this field, yet their conclusions diverge. Some meta-analyses reported that cIV significantly improved net fluid loss (11, 12), whereas others found no difference in either efficacy or adverse events between the two strategies (13). Three major limitations underlie these conflicting results: (i) Sample size limitation: Inadequate sample sizes lack the statistical power to detect clinically hard endpoints. (ii) Marked geographical imbalance: More than 80% of pooled participants were enrolled in European or North American centers, leaving Asian patients severely underrepresented and limiting global generalizability. Asian populations exhibit distinct CYP2C9 and CYP2C19 polymorphisms that alter furosemide metabolism, alongside differential neurohormonal activation and salt-sensitive sodium retention phenotypes. These factors may render continuous infusion—with its stable tubular drug concentrations and attenuated RAAS rebound—particularly advantageous in Asian cohorts (8). (iii) Surrogate efficacy measures: Endpoints such as length of stay or body-weight reduction just “scratch the surface” and fail to convey the extent of dyspnea relief, congestion alleviation, or functional improvement, thereby diminishing the bedside utility of the evidence.

Therefore, we conducted an updated systematic review and meta-analysis that identified and included all randomized controlled trials (RCTs) comparing cIV with iIV furosemide in adult patients with AHF or NYHA class III–IV symptoms.

## Materials and methods

### Study design

This study adhered to the PRISMA guidelines for systematic reviews and meta-analysis (14) and was registered with PROSPERO under the identifier CRD420260223267.

### Search strategy

Two researchers independently searched electronic databases including PubMed, Embase, the Cochrane Library, China National Knowledge Infrastructure (CNKI; https://www.cnki.net), WanFang Data (https://www.wanfangdata.com.cn), and the VIP Database for Chinese Technical Periodicals (CQVIP; https://www.cqvip.com) from their inception to October 1, 2025, for relevant studies. CNKI is the largest comprehensive academic database in China, encompassing journals, dissertations, conference proceedings, and patent literature across all disciplines. WanFang Data is a multidisciplinary Chinese academic repository maintained by the Ministry of Science and Technology, with particular strength in medical journals and institutional repositories. CQVIP (VIP) is a specialized database for Chinese scientific and technical periodicals, providing coverage of biomedical and clinical journals not consistently indexed in international databases. The inclusion of these three Chinese databases was essential to capture RCTs conducted in East Asian populations, which are frequently underrepresented in Western-centric bibliographic resources. The data collected can be accessed through public repositories. The Chinese search terms utilized were “xinshuai,” “xinlishuaijie,” “fusaimi,” “panliniaoji,” “jingmaibengru,” and “chixu.” The corresponding English search terms are presented in Table 1.

**Table 1:** Pubmed search strategy.

Number	Search terms
#1	“heart Failure”[Title/Abstract] OR “acute heart failure”[Title/Abstract] OR “AHF”[Title/Abstract] OR “decompensated heart failure”[Title/Abstract] OR “ADHF”[Title/Abstract]
#2	“furosemide”[Title/Abstract] OR “furosemide”[Title/Abstract] OR “Lasix”[Title/Abstract]
#3	“intravenous infusions”[Title/Abstract] OR “continuous infusion”[Title/Abstract] OR “infusion”[Title/Abstract] OR “IV drip”[Title/Abstract] OR “continuous pump infusion”[Title/Abstract] OR “continuous”[Title/Abstract]
#4	“Randomized Controlled Trial”[Title/Abstract] OR “randomized”[Title/Abstract] OR “RCT”[Title/Abstract]
#5	#1 AND #2 AND #3 AND #4

### Inclusion criteria

The study's eligibility criteria included the following: (i) adults aged ≥18 years diagnosed with AHF classified as NYHA III or IV, regardless of underlying cause, ejection fraction status, or comorbidities; (ii) participants receiving loop diuretics via either continuous intravenous infusion (administered at a constant or titrated rate over >4 h within 24 h) or intermittent bolus dosing (completed within ≤4 h); (iii) primary endpoints: all-cause mortality and freedom from congestion; secondary endpoints: weight loss, 24-h urine volume, length of hospital stay, 72-h urine volume, post-treatment BNP, edema resolution time, time to dyspnea improvement, post-treatment left ventricular ejection fraction (LVEF), and post-treatment left ventricular end-diastolic diameter (LVEDD); and (iv) study designs of randomized controlled trials.

### Exclusion criteria

The exclusion criteria were as follows: (i) individuals with end-stage renal disease (eGFR < 15 mL/min/1.73 m^2^) requiring dialysis (hemodialysis or peritoneal); (ii) concurrent use of IV loop diuretics with hypertonic saline, inotropes, vasoactive agents, or ultrafiltration/renal replacement therapy (RRT), due to potential confounding; (iii) trials employing protocol-based diuretic dosing or delivery modalities aimed at preset urine output goals; (iv) studies without baseline comparability; and (v) studies with unavailable outcomes.

### Study selection

Two review authors independently screened studies for inclusion. We classified the titles and abstracts of all retrieved references as “retrieve” (eligible or potentially eligible/unclear) or “do not retrieve.” Disagreements were resolved by discussion, with a third author available for arbitration if necessary. We obtained full-text reports, and pairs of authors independently assessed the full texts, determined inclusion, and documented reasons for exclusion. Any disagreements were first resolved through discussion; a third author was consulted when required. For records identified in the top-up search, two authors repeated the independent screening process, resolving disagreements in the same manner. Duplicates were identified and removed before and during screening, and multiple reports of the same study were collated so that the unit of interest was the study rather than the publication. The entire selection process was documented in sufficient detail to complete a PRISMA flow diagram and a Characteristics of Included Studies table.

### Data extraction and quality assessment

We developed a structured extraction form and pilot-tested it on at least one included study to ensure field completeness and unambiguous definitions. Three review authors then collaboratively extracted the following variables: fundamental study details (e.g., trial identifier, title, authors, registration number, and publication date), participant demographics (e.g., participant counts for the cIV and iIV arms, patient percentages, NHYA class or LVEF, diagnoses, and inclusion and exclusion criteria), details regarding the intervention (e.g., details of experimental and comparator regimens, permitted concomitant medications, and prohibited medications), and outcomes (e.g., pre-specified primary and secondary endpoints and their measurement time points). Two authors independently extracted outcome data; discrepancies were resolved by consensus, with a third author available for arbitration if required. One author entered the data into Review Manager 5.3, a second author verified accuracy by comparing each field against the extraction form. Finally, a fourth author randomly selected ≥10% of the study characteristic entries and checked them against the original reports to confirm accuracy and consistency. The risk of bias for RCTs was evaluated using the Cochrane Risk of Bias Tool 1.0. In cases of disagreement, a third-party researcher was involved for arbitration. In addition, statistical analyses and the grading of evidence quality were conducted.

### Statistical analysis and evidence quality assessment

All statistical analyses were performed using RevMan 5.3 (The Cochrane Collaboration, Copenhagen, Denmark) and Stata 14 software (StataCorp LLC, College Station, TX, USA). Relative risks (RRs) were determined for dichotomous outcomes, such as all-cause mortality and freedom from congestion. Mean differences (MDs) were used for continuous outcomes, such as weight loss, 24-h urine volume, length of hospital stay, 72-h urine volume, post-treatment BNP, edema resolution time, time to dyspnea improvement, post-treatment LVEF, and post-treatment LVEDD. Heterogeneity across studies was assessed using the chi-squared test and the *I*^2^ statistic. The magnitude of heterogeneity was interpreted according to the Cochrane Handbook: 0%–40% represented low heterogeneity, 30%–60% moderate heterogeneity, 50%–90% substantial heterogeneity, and 75%–100% considerable heterogeneity, with overlapping ranges reflecting uncertainty in these categorizations. A fixed-effects model was applied for studies with *I*^2^ values of 40% or less, suggesting low to moderate heterogeneity. Conversely, a random-effects model was employed for studies with *I*^2^ values above 40%, indicating moderate to high heterogeneity.

In the meta-analysis, whenever *I*^2^ > 50%, we explored the source of heterogeneity by performing subgroup analyses based on region/ethnicity. Trials were stratified into three groups according to the country of enrollment: Western Caucasian (USA, Italy, Spain, and Israel), South/West Asian (India, Pakistan, Turkey, and Egypt), and East Asian (all studies conducted in mainland China).

Sensitivity analyses were initially conducted using the leave-one-out method to identify sources of heterogeneity in efficacy outcomes. Subsequently, a fixed-effects model was reapplied after excluding studies that introduced significant heterogeneity, and the new effect estimate was compared with the original to assess for substantial changes. Publication bias was assessed using Egger's test, with consideration given to results where *p* was 0.05 or lower. The quality of evidence was evaluated across five domains—risk of bias, imprecision, indirectness, publication bias, and inconsistency—using GRADE profiler 3.6, classifying the evidence as high, moderate, low, or very low quality.

### Systematic exploration of heterogeneity sources

We employed a hierarchical approach to identify and address heterogeneity. First, the *I*^2^ statistic and Cochran's *Q-*test were used to quantify inconsistency for each outcome. Second, subgroup analyses stratified by ethnicity (Western Caucasian, South/West Asian, East Asian) were conducted for all outcomes with *I*^2^ > 50%. Third, sensitivity analyses using the leave-one-out method were performed to identify influential studies. Fourth, for outcomes where single-study exclusion markedly reduced heterogeneity, we examined study-specific characteristics (dose, duration, population age) as potential explanatory factors.

### Dealing with missing data

We contacted the study investigators or sponsors to verify key study characteristics and, where possible, to obtain missing numerical outcome data (e.g., when a study was reported only as an abstract). When feasible, we used the Review Manager calculator to derive missing standard deviations (SDs) from other available data—such as confidence intervals—following methods described in the Cochrane Handbook for Systematic Reviews of Interventions. If derivation was not possible and the missing data were judged likely to introduce serious bias, we assessed their impact by repeating the meta-analysis with and without the affected studies.

## Results

### Study selection and characteristics

**Figure 1:**
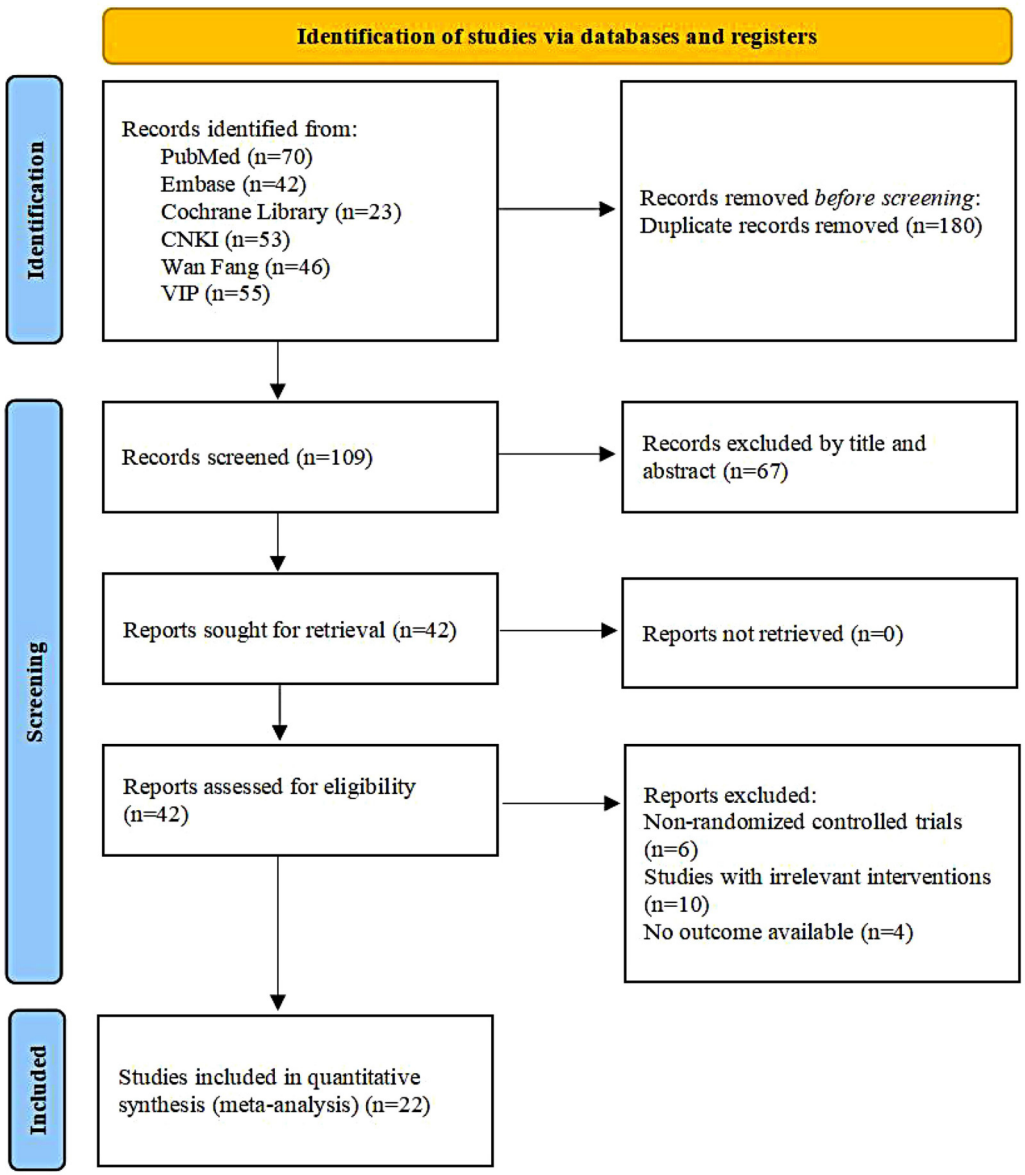
PRISMA diagram of literature search and screening process.

The PRISMA 2020 flow diagram is presented in Figure 1. Of the 289 records initially retrieved, 180 were excluded as duplicates, 67 were excluded based on title/abstract screening (reasons: wrong population (*n* = 24), wrong intervention (*n* = 18), wrong study design (*n* = 19), non-human studies (*n* = 6)), and 20 were excluded after full-text review [reasons: unavailable outcomes (*n* = 4), non-randomized controlled trials (*n* = 6), studies with irrelevant interventions (*n* = 10)]. The final dataset comprised 22 RCTs.

The included 22 RCTs (15–35), were published from 1997 to 2025. A total of 2633 participants were involved. None of the included studies enrolled end-stage renal disease patients (eGFR < 15 mL/min/1.73 m^2^) who were receiving hemodialysis or peritoneal dialysis. The main characteristics of the selected studies are shown in Table 2.

**Table 2:** Main characteristics of the selected studies.

Author	Year	Country	NYHA class or LVEF	Sample size		Sex				Age		Dose of daily furosemide (mg/d)		Duration	Outcomes
				cIV	iIV	cIV		iIV		cIV	iIV	cIV	iIV		
						Male	Female	Male	Female						
Makhoul et al. (15)	1997	Israel	NR	10	10	NR		NR		NR	NR	329 ± 186.7	324 ± 110.8	1 day	24-h urine volume
Allen et al. (16)	2010	USA	LVEF (%) cIV: 31 ± 18 iIV: 39 ± 20	20	21	13	7	9	12	58 ± 16	61 ± 14	162 ± 48	162 ± 52	2 days	All-cause mortality Weight loss 72-h urine volume Length of hospital stay
Thomson et al. (17)	2010	USA	III–IV	26	30	16	10	24	6	56.4 (23–79)	54.6 (18–88)	197 ± 148	172 ± 97	100 h	All-cause mortality Weight loss 24-h urine volume Length of hospital stay
Felker et al. (18)	2011	USA	LVEF (%) cIV: 35 ± 18 iIV: 35 ± 18	152	156	111	41	115	41	65.8 ± 14.1	66.2 ± 13.2	162 ± 48	162 ± 52	3 days	All-cause mortality Freedom from congestion Weight loss 72-h urine volume Length of hospital stay
Llorens et al. (19)	2014	Spain	I–IV	36	iIV1:37 iIV2:36	12	24	iIV1: 12 iIV2: 12	iIV1: 25 iIV2: 24	83 ± 7	iIV1: 83 ± 10 iIV2: 82 ± 8	cIV:240 (10/h)	iIV: 1:80 (20 × 4) iIV: 2:60 (20 × 3)	1 day	24-h urine volume
Palazzuoli et al. (20)	2014	Italy	LVEF (%) cIV: 34.3 ± 10 iIV: 35.8 ± 8	43	39	19	24	21	18	80 ± 4	79 ± 5	170 ± 70	160 ± 80	112 h	All-cause mortality Weight loss 24-h urine volume Length of hospital stay Post-treatment BNP
Shah et al. (21)	2014	India	NR	30	30	23	7	23	7	59.32 ± 13.41	59.27 ± 16.46	100	100 (50 × 2)	2 days	All-cause mortality Length of hospital stay
Yayla et al. (22)	2015	Turkey	LVEF (%) cIV: 41.1 ± 15.7 iIV: 44.8 ± 9.9	15	14	8	7	7	7	65.4 ± 12.2	71.7 ± 10.7	160	160 (80 × 2)	2 days	Weight loss Length of hospital stay
Wang (23)	2015	China	NR	32	34	18	14	20	14	66.1 ± 14.7	68.6 ± 15.4	170 ± 80	188 ± 70	NR	All-cause mortality 24-h urine volume Length of hospital stay Post-treatment BNP
Chen (24)	2016	China	III–IV	57	57	29	28	30	27	62.32 ± 0.29	62.25 ± 0.21	Bolus unknown dose + pump 200 mg 10 mg/h	Bolus unknown dose	NR	Length of hospital stay Edema resolution time Time to dyspnea improvement Post-treatment LVEF
Malkiwodeyar et al. (25)	2017	India	NR	25	25	NR		NR		NR	NR	100	100 (50 × 2)	1 day	All-cause mortality Length of hospital stay
Wang (26)	2017	China	III–IV	30	30	18	12	16	14	67.5 ± 6.2	66.8 ± 6.0	186.9 ± 70.8	172.8 ± 78.5	NR	All-cause mortality Length of hospital stay
Ragab et al. (27)	2018	Egypt	III–IV	20	20	13	7	11	9	53.5 (43.5–62.8)	57 (46–65)	5 mg/h	120 (40 × 3)	1 day	All-cause mortality
Tang (28)	2018	China	IV	40	40	15	25	14	26	45.8 ± 3.2	45.9 ± 2.8	240	240 (120 × 2)	5 days	Length of hospital stay Edema resolution time Time to dyspnea improvement Post-treatment LVEF Post-treatment LVEDD
Wang (29)	2018	China	LVEF (%) cIV: 39 ± 5 iIV: 39 ± 4	31	31	17	14	18	13	69 ± 7	68 ± 7	80–480	80–480	NR	All-cause mortality 24-h urine volume
Liu (30)	2018	China	III–IV	49	49	23	26	25	24	63.4 ± 3.9	64.1 ± 3.8	200	120 (60 × 2)	NR	Length of hospital stay Edema resolution time Time to dyspnea improvement
Ni (31)	2019	China	III–IV	80	80	45	35	43	37	67.92 ± 4.61	67.32 ± 4.47	240	40 (20 × 2)	8 days	Length of hospital stay Edema resolution time Time to dyspnea improvement Post-treatment LVEF Post-treatment LVEDD
Frea (32)	2020	Italy	IV	40	40	37	3	35	5	63.0 ± 13.1	58.7 ± 10.3	198.6 ± 188.0	242.2 ± 176.9	3 days	Freedom from congestion 72-h urine volume
Zheng et al. (10)	2021	China	III–IV	42	39	28	14	25	14	65.53 ± 7.84	67.38 ± 8.57	160/200	160/200	3 days	Freedom from congestion Weight loss 72-h urine volume Length of hospital stay
Hu (33)	2021	China	IV	35	35	23	12	20	15	65.51 ± 1.06	65.47 ± 1.09	200	400 (200 × 2)	7 days	Length of hospital stay Edema resolution time Time to dyspnea improvement Post-treatment LVEF
Khan et al. (34)	2024	Pakistan	LVEF (%) cIV: 44.8 ± 9.9 iIV: 40.5 ± 16.9	420	479	7	8	9	5	71.7 ± 10.7	70.6 ± 8.2	160	160 (80 × 2)	2 days	Weight loss Length of hospital stay
Wang et al. (35)	2025	China	II–IV	34	34	18	16	17	17	58.51 ± 3.57	57.19 ± 3.19	200	240 (120 × 2)	7 days	Length of hospital stay Edema resolution time Time to dyspnea improvement Post-treatment LVEF Post-treatment LVEDD

NR, not reported; cIV, continuous intravenous; iIV, intermittent intravenous. When NYHA class was unavailable, baseline LVEF (mean ± SD) was provided as a surrogate of cardiac function. The two metrics are not pooled or statistically combined; they are displayed separately to maximize transparency.

### Quality assessment of the included studies

**Figure 2:**
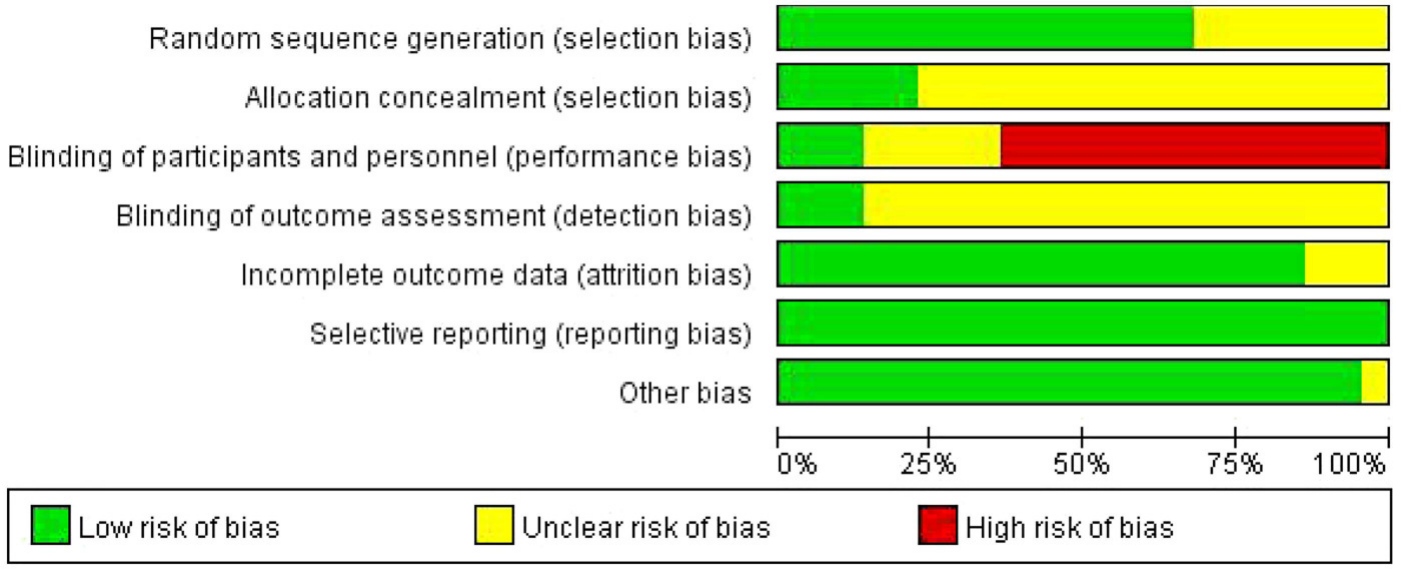
Risk of bias graph.

**Figure 3:**
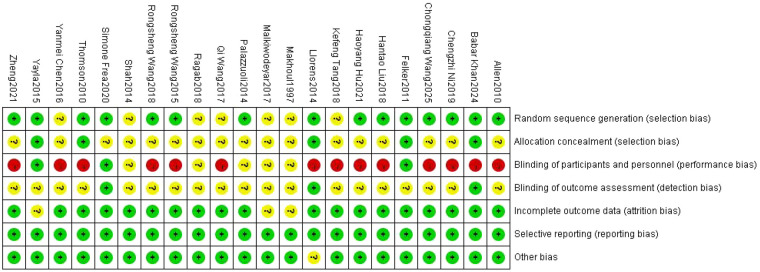
Risk of bias summary. Legend: Green plus sign (+), low risk of bias; yellow minus sign (?), unclear risk of bias; red cross (‒), high risk of bias.

The risk of bias assessment is summarized in Figures 2, 3. Among the 22 included RCTs, adequate random sequence generation was reported in 15 studies (68.1%); allocation concealment was clearly described in only 5 trials (22.7%) (17, 18, 19, 22, 34), with the remaining 17 studies rated as unclear risk due to insufficient methodological reporting. Blinding of participants and personnel was achieved in three trials (13.6%), all of which were conducted in Western countries. Blinding of outcome assessment was reported in three studies (13.6%). Notably, none of the Chinese RCTs employed any form of blinding, contributing to a high risk of performance and detection bias in this subgroup. The overall methodological quality was low to moderate, with particular deficiencies in allocation concealment and blinding domains.

### Primary outcomes

#### All-cause mortality

**Figure 4:**
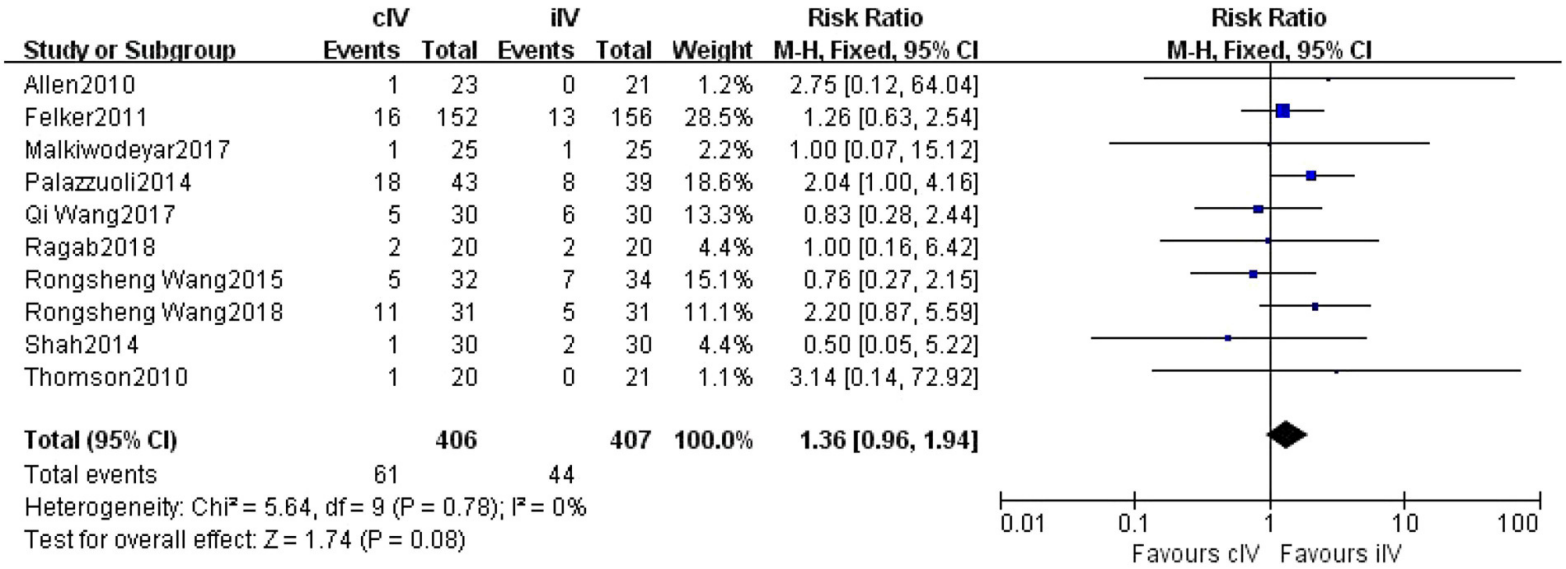
Meta-analysis of all-cause mortality (cIV vs. iIV).

Ten studies (16–18, 20, 21, 23, 25–27, 29) provided data on all-cause mortality for pooling in the meta-analysis. Heterogeneity testing of *P* = 0.78, *I*^2^ = 0%, in all-cause mortality revealed no heterogeneity. The iIV arm showed a numerical trend, compared with the cIV arm, without reaching statistical significance in all-cause mortality (RR: 1.36, 95% CI: 0.96–1.94, *p* = 0.08; Figure 4).

#### Freedom from congestion

**Figure 5:**
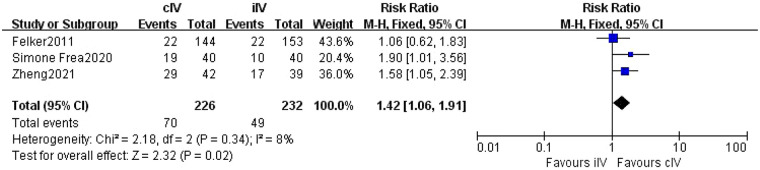
Meta-analysis of freedom from congestion (cIV vs. iIV).

Three studies (10, 18, 32) provided data on freedom from congestion (defined as jugular venous pressure of <8 cm without orthopnea and with trace peripheral edema or no edema). These data were pooled for meta-analysis. Heterogeneity testing of *P* = 0.34, I^2^ = 8%, in freedom from congestion revealed low heterogeneity. The cIV arm showed significant improvements in freedom from congestion compared with the iIV arm (RR: 1.42, 95% CI: 1.06–1.91, *p* = 0.02; Figure 5).

### Secondary outcomes

#### Weight loss (kg)

**Figure 6:**
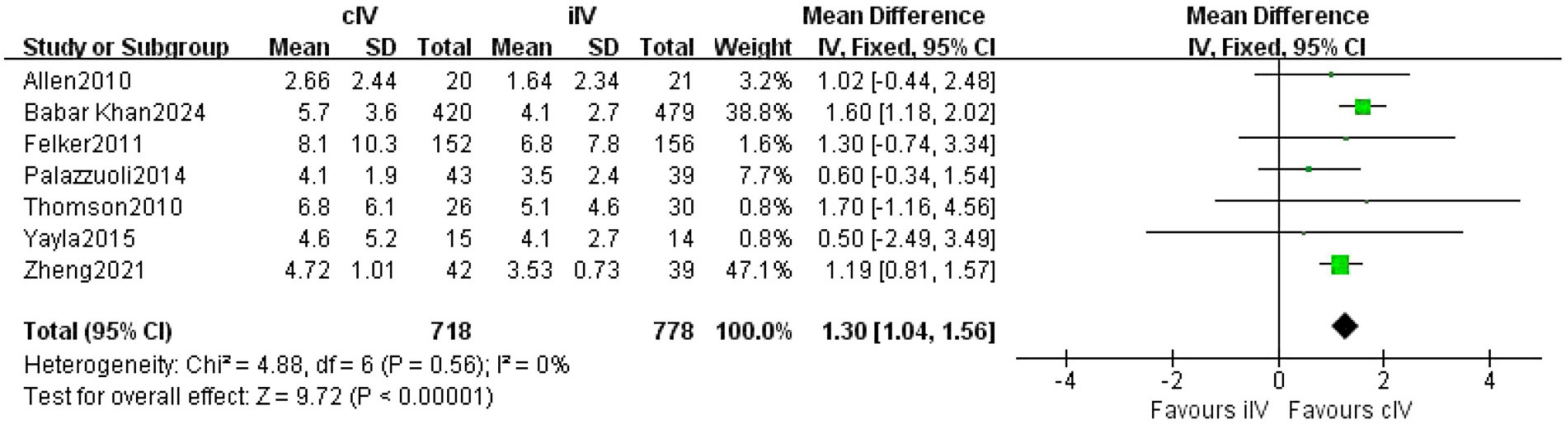
Meta-analysis of weight loss (cIV vs. iIV).

Seven studies (10, 16–18, 20, 22, 34) provided data on weight loss for pooling in the meta-analysis. Heterogeneity testing of *P* = 0.56, I^2^ = 0%, in weight loss showed no heterogeneity. The cIV arm showed significant improvements in weight loss compared with the iIV arm (MD: 1.30, 95% CI: 1.04–1.56, *p* < 0.001; Figure 6).

#### 24-h urine volume (L)

**Figure 7:**
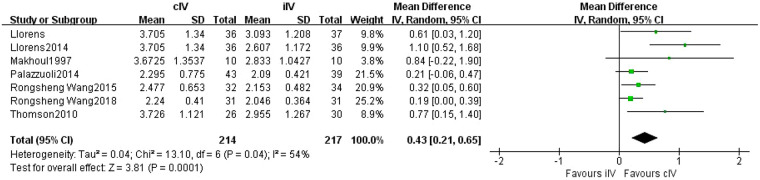
Meta-analysis of 24-h urine volume (cIV vs. iIV). Llorens: Group 1 (continuous intravenous infusion) vs. Group 2 (20 mg each bolus, q6h); Llorens 2014: Group 1 (continuous intravenous infusion) vs. Group 3 (20 mg each bolus, q8h).

Six trials (15, 17, 19, 20, 23, 29) provided data on 24-h urine volume for pooling in the meta-analysis. Heterogeneity testing of *P* = 0.04, I^2^ = 54%, in 24-h urine volume showed high heterogeneity. The cIV arm showed significant improvements in 24-h urine volume compared with the iIV arm (MD: 0.43, 95% CI: 0.21–0.65, *p* < 0.001; Figure 7).

**Figure 8:**
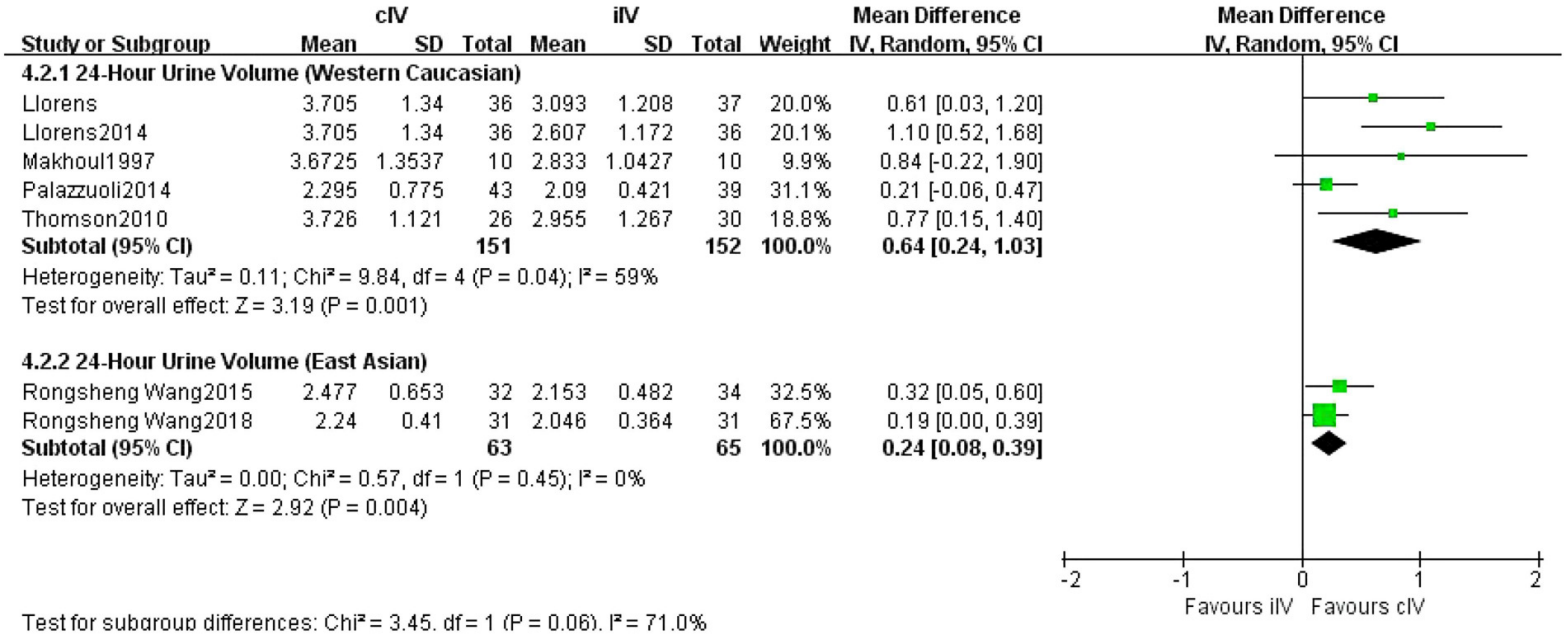
Subgroup analysis of 24-ho urine volume (Western Caucasian and East Asian).

*Subgroup analysis (Western Caucasian and East Asian)*: For the Western Caucasian subgroup, heterogeneity testing of *P* = 0.04, *I*^2^ = 59%, in 24-h urine volume showed high heterogeneity. The cIV arm showed significant improvements in 24-h urine volume compared with the iIV arm (MD: 0.64, 95% CI: 0.24–1.03, *p* = 0.001; Figure 8). For the East Asian subgroup, heterogeneity testing of *P* = 0.45, *I*^2^ = 0%, in 24-h urine volume showed no heterogeneity. The cIV arm showed significant improvements in 24-h urine volume compared with the iIV arm (MD: 0.24, 95% CI: 0.08–0.39, *p* = 0.004; Figure 8).

#### 72-h urine volume (L)

**Figure 9:**
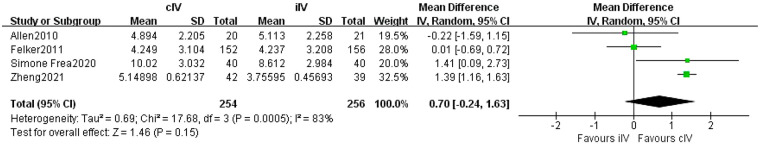
Meta-analysis of 72-h urine volume (cIV vs. iIV).

Four trials (10, 16, 18, 32) provided data on 72-h urine volume for pooling in the meta-analysis. Heterogeneity testing of *P* < 0.001, *I*^2^ = 83%, in 72-h urine volume showed high heterogeneity. The cIV arm showed no significant difference in 72-h urine volume compared with the iIV arm (MD: 0.70, 95% CI: −0.24–1.63, *p =* 0.15; Figure 9).

**Figure 10:**
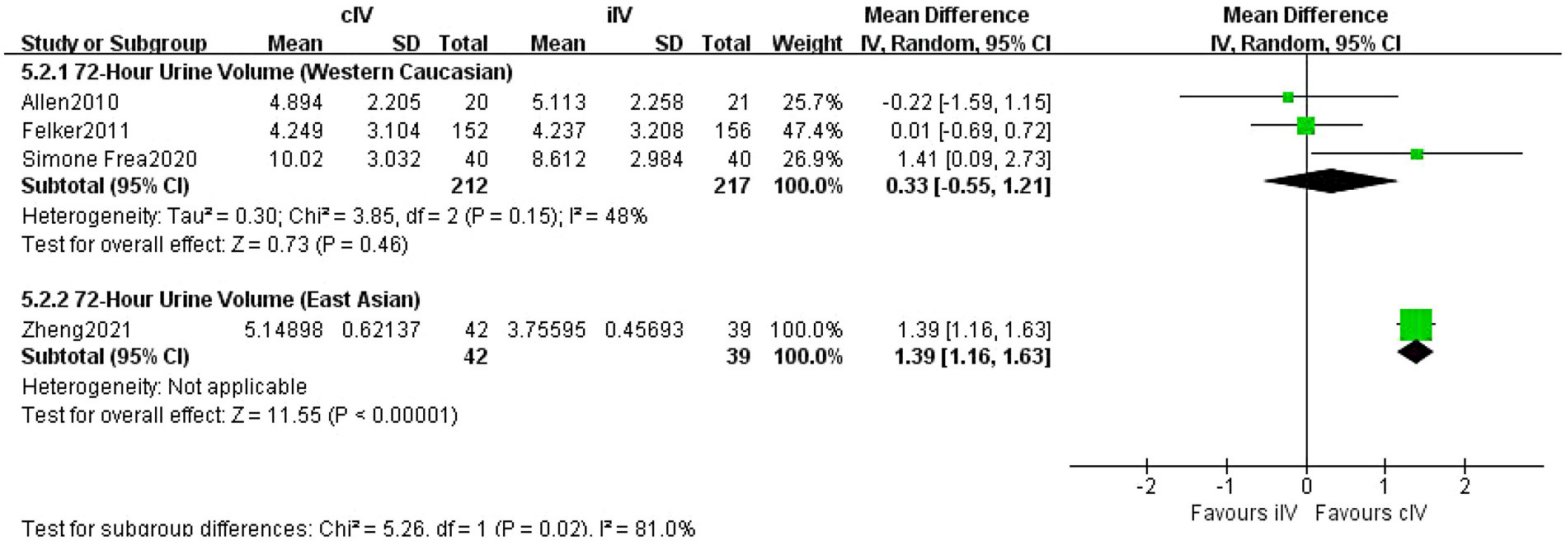
Subgroup analysis of 72-h urine volume (Western Caucasian and East Asian).

*Subgroup analysis (Western Caucasian and East Asian)*: For the Western Caucasian subgroup, heterogeneity testing of *P* = 0.15, *I*^2^ = 48%, in 72-h urine volume showed low heterogeneity. The cIV arm showed no significant difference in 72-h urine volume compared with the iIV arm (MD: 0.33, 95% CI: −0.55–1.21, *p* = 0.15; Figure 10). For the East Asian subgroup, heterogeneity testing was not applicable in the 72-h urine volume. The cIV arm showed significant improvements in 72-h urine volume compared with the iIV arm (MD: 1.39, 95% CI: 1.16–1.63, *p* < 0.001; Figure 10).

### Length of hospital stay (day)

**Figure 11:**
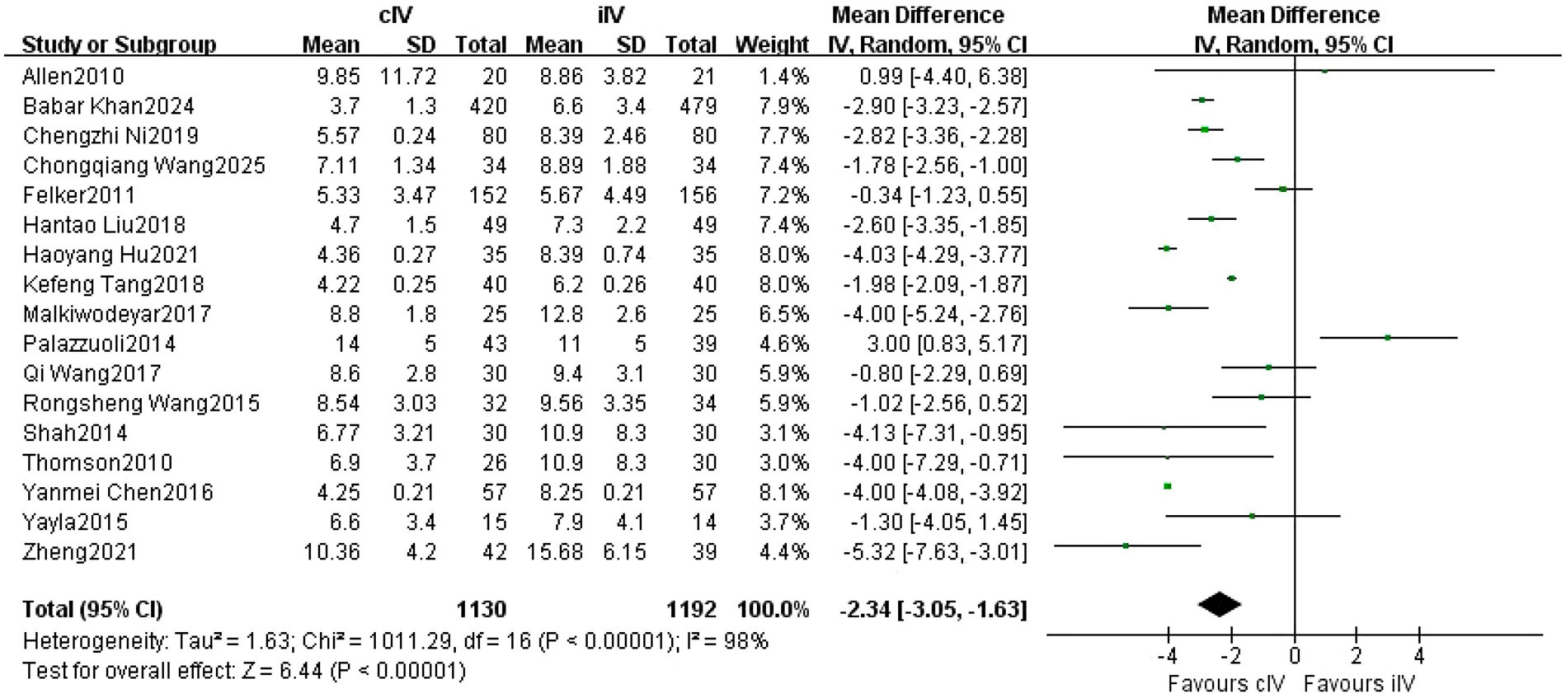
Meta-analysis of length of hospital stay (cIV vs. iIV).

Seventeen trials (10, 16–18, 20–26, 28, 30, 31, 33–35) provided data on length of hospital stay for pooling in the meta-analysis. Heterogeneity testing of *P* < 0.001, *I*^2^ = 98%, in length of hospital stay showed high heterogeneity. The cIV arm showed significant improvements in length of hospital stay compared with the iIV arm (MD: −2.34, 95% CI: −3.05–−1.63, *p* < 0.001; Figure 11).

**Figure 12:**
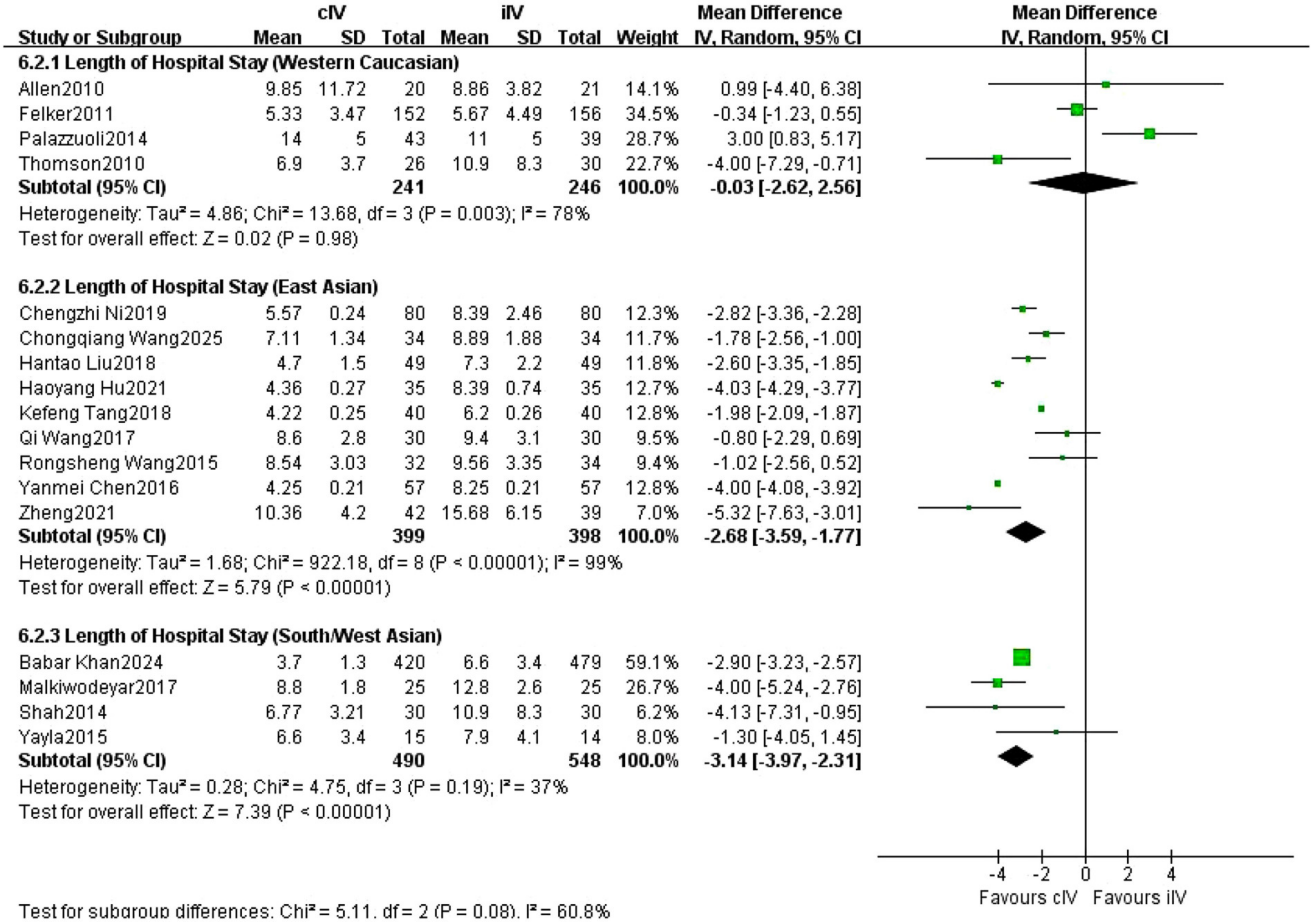
Subgroup analysis of length of hospital stay (Western Caucasian, East Asian, and South/West Asian).

*Subgroup analysis (Western Caucasian and East Asian and South/West Asian)*: For the Western Caucasian subgroup, heterogeneity testing of *P* = 0.003, *I*^2^ = 78%, in length of hospital stay showed high heterogeneity. The cIV arm showed no significant difference in length of hospital stay compared with the iIV arm (MD: −0.03, 95% CI: −2.62–2.56, *p* = 0.98; Figure 12). For the East Asian subgroup, heterogeneity testing of *P* < 0.001, *I*^2^ = 99%, in length of hospital stay showed high heterogeneity. The cIV arm showed significant improvements in length of hospital stay compared with the iIV arm (MD: −2.68, 95% CI: −3.59–−1.77, *p* < 0.001; Figure 12). For the South/West Asian subgroup, heterogeneity testing of *P* = 0.19, *I*^2^ = 37%, in length of hospital stay showed low heterogeneity. The cIV arm showed significant improvements in length of hospital stay compared with the iIV arm (MD: −3.14, 95% CI: −3.97–−2.31, *p* < 0.001; Figure 12).

### Edema resolution time (day)

**Figure 13:**
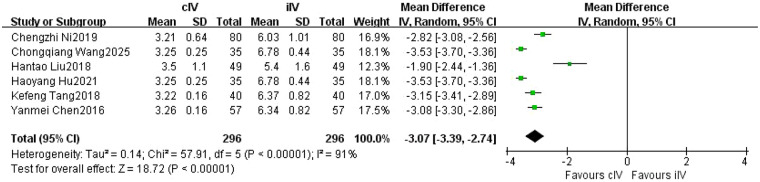
Meta-analysis of length of edema resolution time (cIV vs. iIV).

Six trials (24, 28, 30, 31, 33, 35) provided data on edema resolution time for pooling in the meta-analysis. Heterogeneity testing of *P* < 0.001, *I*^2^ = 91%, in edema resolution time showed high heterogeneity. The cIV arm showed significant improvements in edema resolution time compared with the iIV arm (MD: −3.07, 95% CI: −3.39–−2.74, *p* < 0.001; Figure 13).

### Time to dyspnea improvement (day)

**Figure 14:**
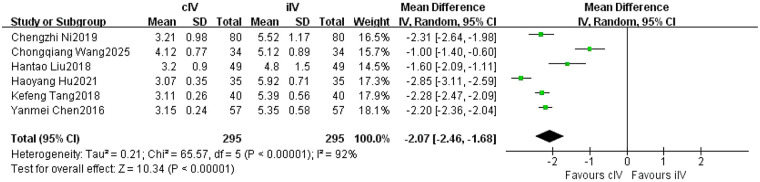
Meta-analysis of time to dyspnea improvement (cIV vs. iIV).

Six trials (24, 28, 30, 31, 33, 35) provided data on time to dyspnea improvement for pooling in the meta-analysis. Heterogeneity testing of *P* < 0.001, *I*^2^ = 92%, in time to dyspnea improvement showed high heterogeneity. The cIV arm showed significant improvements in time to dyspnea improvement compared with the iIV arm (MD: −2.07, 95% CI: −2.46–−1.68, *p* < 0.001; Figure 14).

### Post-treatment LVEF (%)

**Figure 15:**
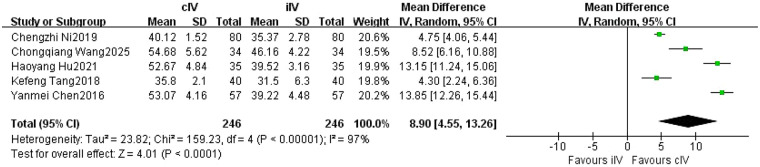
Meta-analysis of post-treatment LVEF (cIV vs. iIV).

Five trials (24, 28, 33, 35, 36) provided data on post-treatment LVEF for pooling in the meta-analysis. Heterogeneity testing of *P* < 0.001, *I*^2^ = 97%, in time to post-treatment LVEF showed high heterogeneity. The cIV arm showed significant improvements in post-treatment LVEF compared with the iIV arm (MD: 8.90, 95% CI: 4.55–13.26, *p* < 0.001; Figure 15).

### Post-treatment LVEDD (mm)

**Figure 16:**
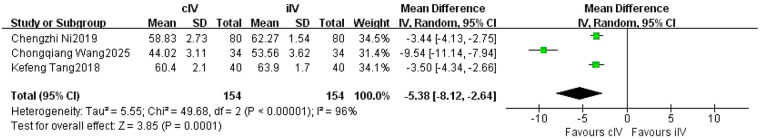
Meta-analysis of post-treatment LVEDD (cIV vs. iIV).

Three trials (28, 31, 35) provided data on post-treatment LVEDD for pooling in the meta-analysis. Heterogeneity testing of *P* < 0.001, *I*^2^ = 96%, in time to post-treatment LVEDD showed high heterogeneity. The cIV arm showed significant improvements in post-treatment LVEF compared with the iIV arm (MD: −5.38, 95% CI: −8.12–−2.64, *p* < 0.001; Figure 16).

### Post-treatment BNP level (pg/mL)

**Figure 17:**
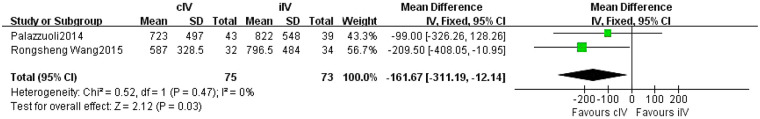
Meta-analysis of post-treatment BNP level (cIV vs. iIV).

Two trials (20, 23) provided data on post-treatment BNP level for pooling in the meta-analysis. Heterogeneity testing of *P* = 0.47, *I*^2^ = 0%, in post-treatment BNP level showed no heterogeneity. The cIV arm showed significant improvements in post-treatment BNP level compared with the iIV arm (MD: −161.67, 95% CI: −311.19–−12.14, *p* = 0.03; Figure 17).

Findings from the meta-analysis are summarized in Table 3.

**Table 3:** Summary of meta-analysis results.

Outcomes		Studies	Participants	Statistical method	Effect estimate	Superior arm
Primary outcomes	All-cause mortality	10	813	Risk ratio (M-H, fixed, 95% CI)	1.36 [0.96, 1.94]	No significant difference
	Freedom from congestion	3	458	Risk ratio (M-H, fixed, 95% CI)	1.42 [1.06, 1.91]	cIV
Secondary outcomes	Weight loss	7	1,496	Mean difference (IV, fixed, 95% CI)	1.30 [1.04, 1.56]	cIV
	24-h urine volume	7	431	Mean difference (IV, random, 95% CI)	0.43 [0.21, 0.65]	cIV
	72-h urine volume	4	510	Mean difference (IV, random, 95% CI)	0.70 [−0.24, 1.63]	No significant difference
	Length of hospital stay	17	2,322	Mean difference (IV, random, 95% CI)	−2.34 [−3.05, −1.63]	cIV
	Edema resolution time	6	592	Mean difference (IV, random, 95% CI)	−3.07 [−3.39, −2.74]	cIV
	Time to dyspnea improvement	6	590	Mean difference (IV, random, 95% CI)	−2.07 [−2.46, −1.68]	cIV
	Post-treatment LVEF	5	492	Mean difference (IV, random, 95% CI)	8.90 [4.55, 13.26]	cIV
	Post-treatment LVEDD	3	308	Mean difference (IV, random, 95% CI)	−5.38 [−8.12, −2.64]	cIV
	Post-treatment BNP level	2	148	Mean difference (IV, fixed, 95% CI)	−161.67 [−311.19, −12.14]	cIV

### Subgroup analyses by ethnicity

To explore potential ethnic-specific treatment effects, we stratified trials into three groups according to the country of enrollment: Western Caucasian (USA, Italy, Spain, and Israel), South/West Asian (India, Pakistan, Turkey, and Egypt), and East Asian (all studies conducted in mainland China).

Western Caucasian subgroup: For 24-h urine volume, the cIV arm showed significant improvements compared with the iIV arm (MD: 0.64 L, 95% CI: 0.24–1.03, *p* = 0.001), though substantial heterogeneity was observed (*I*^2^ = 59%). For 72-h urine volume and length of hospital stay, no significant differences were detected between strategies (MD: 0.33 L, 95% CI: −0.55–1.21, *p* = 0.15; and MD: −0.03 days, 95% CI: −2.62–2.56, *p* = 0.98, respectively).

East Asian subgroup: The cIV arm demonstrated significant improvements across multiple outcomes: 24-h urine volume (MD: 0.24 L, 95% CI: 0.08–0.39, *p* = 0.004; *I*^2^ = 0%), 72-h urine volume (MD: 1.39 L, 95% CI: 1.16–1.63, *p* < 0.001), and length of hospital stay (MD: −2.68 days, 95% CI: −3.59 to −1.77, *p* < 0.001). However, considerable heterogeneity was present for length of hospital stay (*I*^2^ = 99%).

South/West Asian subgroup: The cIV arm showed significant reductions in length of hospital stay (MD: −3.14 days, 95% CI: −3.97 to −2.31, *p* < 0.001) with low heterogeneity (*I*^2^ = 37%). Data for other outcomes in this subgroup were limited.

### Publication bias

Publication bias was assessed for the primary outcomes. Egger's test did not reveal publication bias for all-cause mortality (*t* = −1.75, *p* = 0.534, *p* > 0.05) or freedom from congestion (*t* = −0.02, *p* = 0.858, *p* > 0.05).

### Sensitivity analysis

Sensitivity analyses demonstrated that the direction and magnitude of pooled effect estimates remained stable across statistical models. Switching from a fixed-effects to a random-effects model did not materially alter point estimates or confidence intervals, and no significant differences were observed between model-derived estimates (all *p* > 0.05 for between-model comparisons). Furthermore, the leave-one-out procedure confirmed the robustness of all primary and secondary outcomes. Sequential exclusion of individual studies did not reverse the direction of effect, abolish statistical significance, or produce meaningful changes in heterogeneity, indicating that no single trial exerted undue influence on the pooled results. For the 24-h urine volume outcome, heterogeneity dropped markedly from 54% to 14% after exclusion of the study by Llorens (2014), indicating that this trial was the principal source of inconsistency. We hypothesized that the discrepancy may be attributable to the lower furosemide dose employed in the iIV arm of that study. For the 72-h urine volume outcome, heterogeneity decreased markedly from 83% to 43% after exclusion of the Zheng (2021) study, indicating that this trial was the primary source of inconsistency. We speculated that the discrepancy may be attributable to the relatively low methodological quality of that study. For the post-treatment LVEDD outcome, heterogeneity dropped sharply from 96% to 0% after excluding the study by Chongqiang Wang (2025), indicating that this trial was the sole source of inconsistency. We hypothesized that the discrepancy may be attributable to the younger age of the enrolled participants and the longer duration of intervention in that study. For the four outcomes of length of hospital stay, edema resolution time, time to dyspnea improvement, and post-treatment LVEF, leaving out any single study did not materially reduce heterogeneity. We attributed this to the following sources of systematic dispersion: (1) Length of hospital stay: “Zero time” and endpoints were inconsistently defined: some trials counted from randomization, others from first diuretic administration. Discharge was recorded as “actual,” “medical decision,” or “24 h after clinical stability.” These discrepancies could introduce 0.5–1.5-day systematic differences across studies. (2) Edema resolution time and time to dyspnea improvement: Both rely on subjective assessment. Edema resolution was usually graded daily at the bedside without support from imaging or bio-impedance. Dyspnea improvement was typically based on patient-reported “significant relief” using variable scales. Large inter-rater/inter-patient variability inflated random error and amplified true heterogeneity. (3) Post-treatment LVEF: Inter-center heterogeneity in ultrasound machines, transducer frequencies, image acquisition protocols, and measurement algorithms, together with operator-dependent habits, produced systematic deviations in the same patient, constituting a persistent source of between-study variance. All forest plots of sensitive analyses are shown in Supplementary file S1.

### Evidence quality assessment

All outcomes were assessed using the GRADE approach. Risk bias: All outcomes were judged to have a serious risk due to unclear allocation concealment and lack of blinding in the included studies. Inconsistency: Low heterogeneities were found in four outcomes due to which they were considered to have no risk of inconsistency. Indirectness: No outcome was judged to have significant indirectness because all studies were direct comparisons. Imprecision: The outcomes of all-cause mortality and 72-h urine volume were considered at serious risk of imprecision due to insufficient sample sizes. Publication bias: No outcome exhibited publication bias. Overall: The outcome of 72-h urine volume displayed very-low-quality evidence. The outcomes of all-cause mortality, 24-h urine volume, length of hospital stay, edema resolution time, time to dyspnea improvement, post-treatment LVEF, and post-treatment LVEDD had low-quality evidence. The outcomes of freedom from congestion, weight loss, and post-treatment BNP level had moderate-quality evidence. Evidence quality assessments are shown in Table 4.

**Table 4:** GRADE evidence profiles of outcomes.

Outcome	Number of studies	Assessment of evidence quality					Number of participants	Effect (95%CI)	Evidence quality
		Risk bias	Inconsistency	Indirectness	Imprecision	Publication bias			
All-cause mortality	10	Serious	No	No	Serious	Undetected	813	1.36 [0.96, 1.94]	Low
Freedom from congestion	3	Serious	No	No	No	Undetected	671	1.42 [1.06, 1.91]	Moderate
Weight loss	7	Serious	No	No	No	Undetected	1,496	1.30 [1.04, 1.56]	Moderate
24-h urine volume	6	Serious	Serious	No	No	Undetected	431	0.43 [0.21, 0.65]	Low
72-h urine volume	4	Serious	Serious	No	Serious	Undetected	510	0.70 [−0.24, 1.63]	Very Low
Length of hospital stay	17	Serious	Serious	No	No	Undetected	2,322	−2.34 [−3.05, −1.63]	Low
Edema resolution time	6	Serious	Serious	No	No	Undetected	598	−3.07 [−3.39, −2.74]	Low
Time to dyspnea improvement	6	Serious	Serious	No	No	Undetected	590	−2.07 [−2.49, −1.68]	Low
Post-treatment LVEF	5	Serious	Serious	No	No	Undetected	492	8.90 [4.55, 13.26]	Low
Post-treatment LVEDD	3	Serious	Serious	No	No	Undetected	308	−5.38 [−8.12, −2.64]	Low
Post-treatment BNP level	2	Serious	No	No	No	Undetected	148	−161.67 [−311.19, −12.14]	Moderate

## Discussion

This systematic review and meta-analysis of 22 randomized controlled trials, comprising 2,633 patients with AHF, compared the efficacy of continuous intravenous infusion with intermittent intravenous bolus furosemide. The results demonstrated that cIV was significantly superior to iIV in several clinical endpoints (freedom from congestion, weight loss, length of hospital stay, time to edema resolution, and time to dyspnea improvement) and surrogate endpoints (post-treatment LVEF, LVEDD, and BNP levels). However, there were no statistically significant differences between the two groups in all-cause mortality or 72-h urine output.

The absence of a significant difference in all-cause mortality between the two strategies can be attributed to two main factors: First, the overall number of death events was small (105 cases across 10 studies), yielding insufficient statistical power. Second, cIV primarily targets the early “congestion-dominant” pathological trajectory, whereas acute heart failure deaths also arise from non-congestive components such as infection, sudden cardiac death, and renal failure, diluting any potential survival benefit. In contrast, cIV was significantly superior to iIV in achieving congestion-free status (RR = 1.42, *p* = 0.02), shortening time to edema resolution (MD = −3.07 days, *p* < 0.001), and accelerating dyspnea improvement (MD = −2.07 days, *p* < 0.001), indicating that continuous infusion rapidly relieves fluid overload and promptly enhances both objective indices and patients’ subjective comfort and tolerability. These advantages were underpinned by cIV's ability to produce greater 24-h urine output (MD = 0.43 L, *p* < 0.001) and weight reduction (MD = 1.30 kg, *p* < 0.001), leading to earlier achievement of optimal volume depletion; although cumulative urine output at 72 h did not differ significantly, the early negative fluid balance was sufficient to alleviate congestion. Mechanistically, cIV maintains steady drug concentrations at the renal tubular site, avoiding the “peak-and-trough” fluctuations and post-receptor rebound seen with iIV. This reduces repeated activation of the renin–angiotensin–aldosterone system (RAAS) and sympathetic nervous system, thereby creating a more stable neurohormonal environment that enhances natriuresis (37, 38). In terms of cardiac function, cIV was associated with a markedly greater increase in LVEF (MD = 8.90%, *p* < 0.001), a larger reduction in LVEDD (MD = −5.38 mm, *p* < 0.001), and a more pronounced decrease in BNP (MD = −161.67 pg/mL, *p* = 0.03), suggesting that sustained preload reduction improved hemodynamics and promoted myocardial recovery. However, LVEF, LVEDD, and BNP are surrogate endpoints that reflect physiological changes but do not directly measure patient-centered clinical outcomes such as mortality, quality of life, or functional capacity. While improvements in these biomarkers and imaging parameters are encouraging, they cannot be equated with proven clinical benefit. Moreover, the superiority claims for post-treatment LVEF (5 trials, *n* = 492), LVEDD (3 trials, *n* = 308), and BNP (2 trials, *n* = 148) are based on small numbers of included trials with limited sample sizes, reducing the precision and generalizability of these estimates. The considerable heterogeneity observed for LVEF (*I*^2^ = 97%) and LVEDD (*I*^2^ = 96%) further underscores the uncertainty surrounding these surrogate findings. In contrast, length of hospital stay was shortened by 2.34 days (*p* < 0.001), lowering healthcare costs and the risk of nosocomial infections, thereby delivering both economic and clinical benefits. Although continuous furosemide infusion demonstrated favorable effects on 24-h and 72-h urine output, length of stay, time to edema resolution, time to dyspnea improvement, post-treatment LVEF, and LVEDD, the marked between-study heterogeneity (*I*^2^ > 50%) unavoidably downgraded the overall quality of evidence. Moreover, the pooled reduction in BNP levels achieved statistical significance; however, this finding was based on only two trials, one of which detected no inter-group difference. Consequently, the BNP outcome should be regarded as exploratory and requires further validation.

Cost-effectiveness considerations: The clinical advantages of continuous intravenous infusion must be weighed against its economic implications. Continuous infusion requires additional immediate expenditures, including infusion pump equipment, dedicated intravenous lines, and more intensive nursing monitoring for dose titration and adverse event surveillance. These incremental costs may be particularly relevant in resource-limited settings. However, the observed 2.34-day reduction in length of hospital stay (moderate heterogeneity across 17 trials, *n* = 2,322) represents a substantial counterbalancing economic benefit. Hospital bed-days constitute a major driver of healthcare expenditure in acute heart failure management, and even modest reductions can yield meaningful system-level cost savings. Furthermore, faster resolution of congestion and dyspnea may translate into reduced need for adjunctive therapies, lower nosocomial infection risk, and earlier return to functional independence, generating additional indirect economic value. Formal cost-effectiveness analyses—incorporating country-specific hospitalization costs, nursing staff ratios, and infusion pump procurement expenses—are needed to quantify the net economic impact and inform reimbursement policy. Such analyses should adopt a healthcare system perspective and account for heterogeneity in resource availability across Western, East Asian, and South/West Asian settings, given the differential magnitude of length-of-stay reduction observed across ethnic subgroups in our analysis.

To explore the sources of heterogeneity, we conducted subgroup analyses by ethnicity. For 72-h urine volume, heterogeneity dropped markedly within each ethnic subgroup, indicating that racial differences were a major contributor to the observed inconsistency. For other outcomes, heterogeneity remained unchanged after stratification, leaving the sources of heterogeneity unidentified. In the sensitivity analyses, we employed a leave-one-out approach for all outcomes. Removal of any single study did not alter the direction of the pooled effect or abolish statistical significance. Switching from a fixed-effects to a random-effects model left the point estimate of the relative risk virtually unchanged, indicating that no individual trial exerted excessive influence. Further analysis after excluding studies at high risk of bias continued to show consistent effect directions, demonstrating resilience of conclusions to differences in study quality. The GRADE evaluation rated the evidence for all-cause mortality as “low quality,” predominantly because of the small number of events (105 deaths) and limitations in allocation concealment and blinding across several trials. No indirectness or publication bias was detected, but serious imprecision warranted downgrading. In contrast, congestion-free status was judged “moderate quality.” Despite some risk of bias, the effect was consistent, heterogeneity was low (*I*^2^ = 8%), the outcome was direct and reproducible, and downgrading to “high” was only precluded by the limited sample size. Overall, the outcome of 72-h urine volume was rated as very low-quality evidence, while the outcomes of all-cause mortality, 24-h urine volume, length of hospital stay, edema resolution time, time to dyspnea improvement, post-treatment LVEF, and post-treatment LVEDD were supported by low-quality evidence. However, the outcomes of freedom from congestion, weight loss, and post-treatment BNP level were supported by moderate-quality evidence. Taken together, the sensitivity analyses confirmed the stability of our main results, while the GRADE approach indicated that cIV offers moderate certainty of benefit in relieving congestion. However, evidence for a mortality advantage remained insufficient, emphasizing the need for clinical decisions to integrate patient phenotype and individualized goals.

Previous studies have laid an important foundation for this field, yet their conclusions often point in opposite directions. Rasoul et al. (13) concluded that limited sample sizes and heterogeneous outcomes preclude declaring either strategy superior overall. Ng and Yap (11) showed that cIV outperformed iIV in diuretic effect and BNP reduction, although all-cause mortality, length of stay, and electrolyte disturbance rates were identical. Chan et al. (12) further demonstrated that cIV increased daily urine output and promoted weight loss. Kuriyama et al. emphasized that, although cIV did not shorten hospitalization or lower mortality, it conferred a definite 0.63-kg advantage in weight reduction without increasing adverse events. Huang et al. (36), pooling a larger sample, found cIV significantly superior in weight loss (MD 1.08 kg) and 24-h urine volume (MD 335 mL) to iIV, whereas hospital stay and mortality remained comparable. Kuriyama et al. (39) reported that compared with intermittent dosing, continuous infusion of furosemide was not associated with improvements in all-cause mortality, length of hospital stay, or 24-h urine output, but it was significantly associated with greater weight loss. There were no differences in hypokalemia, hyponatremia, elevated serum creatinine, or hypotension. Compared with intermittent administration, continuous infusion of furosemide was associated with greater weight loss and a potential increase in 24-h urine output. Our findings differ somewhat from the recent Cochrane review by Rasoul et al. (13), which concluded that continuous infusion may result in little to no difference compared with bolus injection across all measured outcomes. Several methodological distinctions account for this discrepancy. First, the Cochrane review applied stringent exclusion criteria, thereby reducing the eligible pool to seven RCTs (*n* = 681). By contrast, our broader inclusion strategy captured 22 RCTs (*n* = 2,633), incorporating a substantial proportion of trials from East Asia—particularly China—that were excluded from or unavailable to the Cochrane analysis, substantially expanding the sample size and supplementing non-Caucasian data. Second, the Cochrane review reported very low to low certainty of evidence for all outcomes, primarily due to risk of bias and imprecision; sensitivity analyses excluding high-risk trials further attenuated effect estimates toward the null. Our analysis likewise employed sensitivity analyses and GRADE assessment, with principal conclusions remaining stable after single-study exclusion, indicating a degree of robustness. Third, the Cochrane review systematically evaluated adverse events, finding no significant difference in acute kidney injury (RR 1.02, 95% CI 0.70–1.49), electrolyte disturbances, or ototoxicity between strategies—a safety dimension that our analysis could not address quantitatively due to incomplete reporting in the primary studies, although this limitation has been acknowledged in our discussion. Taken together, the two reviews adopted different inclusion strategies and population bases: The Cochrane review found no significant difference in Western populations with strictly controlled confounders, whereas our analysis observed advantages of continuous infusion in relieving congestion within a broader, more ethnically diverse sample. This divergence likely reflects differences in patient phenotypes, dosing practices, and healthcare systems across regions rather than a fundamental contradiction. Large-scale, multicenter, methodologically rigorous RCTs are warranted to validate these findings. Nevertheless, previous studies suffered from three non-negligible shortcomings: (i) inadequate sample sizes lacking the statistical power to detect clinically meaningful hard endpoints; (ii) marked geographical imbalance—more than 80% of pooled participants were enrolled in European or North American centers, leaving Asian patients severely underrepresented and limiting global generalizability; and (iii) endpoints like shorter hospital stays or weight loss fail to capture what matters most to patients—whether their breathing gets easier, their swelling goes down, or they can walk farther and do more.

This study systematically addressed prior shortcomings in three key ways: (i) expanding the sample to 22 randomized controlled trials totaling 2,633 patients, markedly increasing power to detect hard endpoints; (ii) incorporating a substantial number of trials from Asian countries—especially China—thereby correcting the previous underrepresentation of these populations; and (iii) adopting a patient-centered approach that added clinically meaningful outcomes such as congestion-free rate, time to edema resolution, and time to dyspnea improvement to traditional efficacy measures, ensuring the evidence reflects real-world benefits at the bedside. Nevertheless, this study had several evident limitations. First, the number of hard endpoint events (all-cause mortality) was still small, providing insufficient power to detect potentially clinically important differences. Second, most of the included RCTs were of low methodological quality, lacking blinding and with unclear allocation concealment, introducing a non-negligible risk of bias. Large-scale, multicenter, rigorously designed randomized controlled trials—with adequate blinding and concealment—are urgently needed to clarify the true impact of continuous infusion on hard endpoints and long-term prognosis. Third, because none of the included RCTs reported CKD prevalence, baseline eGFR, or concomitant drug regimens, we could not perform subgroup analyses or meta-regressions for renal function or drug interactions. This absence represented a major limitation of the current evidence base and underscores the need for more comprehensive phenotyping in future diuretic trials. Fourth, although the inclusion of Chinese databases expanded the sample size and supplemented non-Caucasian data, it also introduced new sources of bias, including differences in patient age, body weight, comorbidity profiles, drug dosages, and laboratory testing standards. In addition, adverse events, including acute kidney injury (AKI), electrolyte disturbances, and ototoxicity, were not systematically evaluated in this meta-analysis, representing a significant limitation that warrants attention. Although continuous infusion demonstrated superiority in relieving congestion, its safety profile remains inadequately characterized. Palazzuoli et al. reported a higher incidence of renal function deterioration in the continuous infusion group compared with intermittent bolus, possibly attributable to sustained high tubular concentrations leading to renal medullary ischemia and pre-renal hypoperfusion. Similar trends were observed in some Chinese studies. Moreover, high-dose or prolonged furosemide use may precipitate electrolyte imbalances (e.g., hypokalemia, hyponatremia, hypomagnesaemia) and ototoxicity; these risks may be amplified under continuous infusion because drug concentrations are maintained at elevated levels for extended periods. Unfortunately, the Chinese studies included in this analysis provided insufficient or inconsistent reporting of adverse events, precluding quantitative synthesis. Future trials should prospectively document AKI incidence, serial electrolyte monitoring, and audiological assessments to comprehensively balance the benefits and risks of continuous infusion, thereby informing individualized clinical decision-making.

The clinical implications of our findings should be interpreted within a patient-centered, individualized framework. For patients with diuretic resistance—characterized by prior requirements for high-dose loop diuretics or inadequate 24-h urine output—continuous infusion may be preferable, as sustained tubular drug concentrations circumvent peak-and-trough fluctuations and potentially overcome pharmacodynamic resistance. Conversely, in patients with baseline renal impairment, clinicians must carefully balance the benefits of rapid decongestion against the risk of renal function deterioration; enhanced monitoring of serum creatinine and electrolytes is warranted in this subgroup. For hemodynamically unstable patients or those requiring tight volume control (e.g., severe hyponatremia), the stable pharmacokinetic profile of continuous infusion may offer distinct advantages over intermittent boluses. Future trials should employ phenotypic stratification to identify optimal diuretic strategies for distinct patient subgroups, thereby moving beyond a “one-size-fits-all” approach toward precision-guided diuretic therapy.

In conclusion, our study indicated that, compared with intravenous bolus furosemide, continuous infusion was associated with faster relief of congestion, greater weight loss, and greater reduction in post-treatment BNP levels (moderate-quality evidence). However, no significant advantage was demonstrated for the remaining outcomes or long-term survival, as judged by the quality of evidence and effect estimates.

## Conclusions

Compared with intravenous bolus furosemide, continuous infusion was associated with faster relief of congestion, greater weight loss, and greater reduction in post-treatment BNP levels (moderate-quality evidence). However, no significant advantage was demonstrated for the remaining outcomes or long-term survival, as judged by the quality of evidence and effect estimates.

## Data Availability

The original contributions presented in the study are included in the article/Supplementary Material; further inquiries can be directed to the corresponding author/s.
